# Eight-month angiographic outcomes and in-stent restenosis in patients undergoing percutaneous coronary intervention on unprotected left main coronary artery

**DOI:** 10.1097/MCA.0000000000001557

**Published:** 2025-10-24

**Authors:** Mauro Massussi, Andrea Drera, Edoardo Pancaldi, Elisa Pezzola, Luca Tagazzini, Claudia Fiorina, Luca Branca, Giuliano Chizzola, Marco Metra, Salvatore Curello, Marianna Adamo

**Affiliations:** a Department of Medical and Surgical Specialties, Radiological Sciences, and Public Health, University of Brescia; b Cardiac Catheterization Laboratory and Cardiology, Department of Cardiac and Thoracic surgery, ASST Spedali Civili di Brescia; c Faculty of Medicine and Surgery, University of Brescia, Brescia, Italy

**Keywords:** in-stent restenosis, left main coronary artery, percutaneous coronary intervention

## Abstract

In-stent restenosis (ISR) remains a significant complication of percutaneous coronary intervention (PCI) for left main coronary artery (LMCA) disease, with potentially severe consequences. This study aimed to evaluate the incidence and predictors of ISR and highlight the role of systematic angiographic follow-up in optimizing patient outcomes. We conducted a retrospective cohort study including 229 patients who underwent LMCA PCI between 2013 and 2023 at ASST Spedali Civili di Brescia. All patients underwent systematic angiographic follow-up at 8 months. Data on clinical, angiographic, and procedural characteristics were collected and analyzed using univariate and multivariate logistic regression to identify predictors of ISR. Kaplan–Meier survival analysis was employed to assess outcomes. ISR was identified in 24 patients (10.5%) during angiographic follow-up, with 29.2% being symptomatic. Chronic kidney disease (CKD; odds ratio: 3.84, *P* = 0.003) and diabetes (odds ratio: 3.18, *P* = 0.008) emerged as independent predictors of ISR. Multivariate analysis confirmed these associations. Survival rates were high, with 97.7% at 1 year, 92.2% at 2 years, and 81.5% at 4 years. Subanalyses showed trends toward higher mortality among patients with CKD or diabetes but no significant differences between patients with acute and chronic coronary syndromes. In conclusion, ISR remains a clinically significant challenge after LMCA PCI, with CKD and diabetes as key predictors. Systematic angiographic follow-up is essential for early ISR detection, especially in high-risk populations, as the majority of cases are asymptomatic. These findings emphasize the need for tailored surveillance strategies to improve outcomes.

## Introduction

Management of left main coronary artery (LMCA) disease remains one of the most challenging areas in interventional cardiology [1]. According to European Society of Cardiology guidelines, coronary artery bypass grafting (CABG) is the preferred strategy for most patients; however, percutaneous coronary intervention (PCI) is an appropriate alternative in cases of low to intermediate anatomical complexity [2]. The evaluation of XIENCE versus coronary artery bypass surgery for effectiveness of left main revascularization trial demonstrated comparable outcomes between PCI with second-generation drug-eluting stents (DES) and CABG in terms of the composite endpoint of death, stroke, or myocardial infarction at 3 years [15.4% vs. 14.7%; *P* = 0.02 for noninferiority; hazard ratio: 1.00; 95% confidence interval (CI): 0.79–1.26; *P* = 0.98 for superiority] [3]. Despite technological advances, in-stent restenosis (ISR) remains a clinically significant complication of PCI, particularly in LMCA, given its critical role in myocardial perfusion [4–6]. ISR in LMCA stents can have catastrophic consequences if undiagnosed or untreated. Noninvasive imaging techniques, such as stress echocardiography, cardiac magnetic resonance, or single-photon emission computed tomography (CT), may be unreliable due to the extensive myocardial mass at risk, leading to attenuation of diagnostic accuracy [7]. Coronary CT is also suboptimal for ISR evaluation due to blooming artifacts from calcifications. In this context, angiographic follow-up emerges as a valuable tool for early detection of ISR, especially given the high risk of silent restenosis in these patients [8]. This study aims to evaluate the incidence and predictors of ISR in patients undergoing PCI for LMCA disease and to highlight the critical role of angiographic follow-up in identifying ISR and optimizing patient outcomes.

## Methods

This retrospective cohort study included patients diagnosed with LMCA disease who underwent PCI at the Hemodynamics Laboratory of ASST Spedali Civili di Brescia between 2013 and 2023. All patients underwent scheduled angiographic follow-up at 8 months post-PCI as part of our institutional protocol for unprotected LMCA PCI, based on evidence suggesting that the incidence of ISR peaks between 6 and 12 months postprocedure [9]. The angiographic follow-up protocol included selective LMCA cannulation only (excluding the right coronary artery if no significant disease was reported) and at least two complementary angiographic projections to minimize contrast media use and reduce the risk of kidney impairment. Patients with a prior history of CABG involving the left anterior descending or left circumflex coronary artery were excluded from the study.

Clinical, angiographic, and echocardiographic data were collected from a dedicated database through the review of discharge records and coronary angiographies. Information was retrieved from the Milos and SUITESTENSA data management systems used at ASST Spedali Civili di Brescia. For each patient, angiographic parameters included lesion classification according to the Medina system, presence of calcifications, multivessel coronary artery disease, number of stents deployed, and procedure duration. PCI techniques were categorized as: Provisional PCI or DK crush technique. Follow-up angiographic data were collected via the SUITESTENSA system. Mortality data and date of death were obtained from the Integrated Health System software. The study complied with the Declaration of Helsinki and was approved by the local scientific and ethical committee. The following outcomes were defined: Primary outcomes as the presence of ISR at 8-month follow-up angiography. ISR is defined as a luminal obstruction >50% within the stented segment or within 5 mm proximal or distal to the stent edges [10]. If the stenosis is >70%, ISR is confirmed angiographically. For stenoses between 50 and 70%, further assessment was performed using physiological evaluation with fractional flow reserve (FFR, cutoff <0.80 with adenosine infusion) to determine hemodynamic severity. In cases of multiple tandem stenoses, where pressure pull-back alone could not accurately define lesion significance, intravascular ultrasound (IVUS) was used as an alternative for a more detailed morphological assessment [11,12].

Secondary outcomes included all-cause mortality and the presence of angina symptoms at follow-up, classified according to the Canadian Cardiovascular Society (CCS) scale.

Continuous variables were expressed as mean ± SD, while categorical variables were presented as absolute values and percentages. Event-free survival during follow-up was represented using Kaplan–Meier curves. The log-rank test (Mantel-Cox) was used to assess statistical significance between survival curves. Finally, logistic regression models were employed to identify predictors of ISR at 8-month angiographic follow-up. Both univariate and multivariate logistic regression analyses were conducted, with ISR as the dichotomous outcome variable. All analyses were performed using STATA software (StataCorp LLC, College Station, Texas, USA).

### Missing data handling

Baseline variables such as age, sex, BMI, diabetes, and chronic kidney disease (CKD) were fully available for all patients, as they were verified through laboratory results and electronic medical records during hospitalization. Information regarding angina status, acute coronary syndrome (ACS) vs. CCS classification, and previous PCI or CABG was validated via a comprehensive review of procedural reports and coronary angiograms by two independent operators (M.M. and A.D.). Procedural variables (e.g. Medina classification, stenting strategy, use of IVUS/FFR, and mechanical circulatory support) were likewise derived from direct review of angiographic recordings and catheterization lab documentation. For variables such as smoking status, dyslipidemia, peripheral artery disease (PAD), and atrial fibrillation, data were primarily retrieved from discharge summaries and clinical history. In these cases, the possibility of underreporting due to incomplete documentation cannot be entirely excluded. Nevertheless, missing data were rare and randomly distributed. Therefore, no imputation was performed.

## Results

**Fig. 1:**
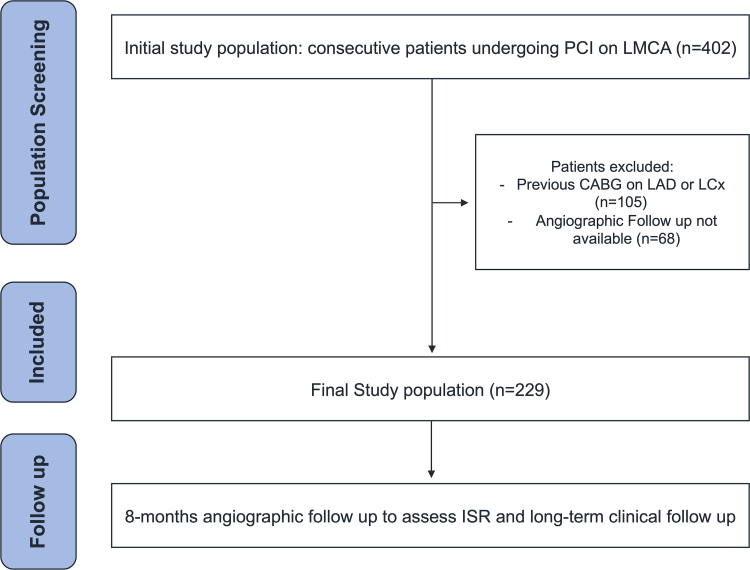
Flowchart illustrating the patient selection and enrollment process in the study. CABG, coronary artery bypass grafting; ISR, in-stent restenosis; LAD, left anterior descending artery; LCx, left circumflex artery; LMCA, left main coronary artery; PCI, percutaneous coronary intervention.

The initial cohort of patients enrolled in the study consisted of 402 individuals. From this group, all patients with a history of CABG involving either the left anterior descending or the left circumflex coronary artery (*n* = 105), as well as those who did not undergo an angiographic follow-up at 8 months (*n* = 68), were excluded, resulting in a final study population of 229 patients (Fig. 1). The baseline characteristics and reasons for exclusion of the 68 patients are provided in Supplementary Table 1, Supplemental digital content 1, https://links.lww.com/MCA/A768. As detailed in Table 1, the median age of the study population was 69 years (interquartile range, 60–77 years). Most patients were male (78.2%). Regarding major cardiovascular risk factors, nearly all patients had dyslipidemia (89.5%), while 26.2% had diabetes. Additionally, 24.9% were active smokers at the time of the procedure, and 20.9% had severely impaired renal function, defined as a creatinine clearance below 30 ml/min.

**Table 1:** Baseline characteristics of the study population

Total population (*N* = 229)	
Baseline variables	
Age (years)	69 (60–77)
Males, *n* (%)	179 (78.2)
LVEF (%)	58 (20–67)
BMI (kg/m²)	26.12 (16.65–28.4)
Smoking, *n* (%)	57 (24.9)
Dyslipidemia, *n* (%)	205 (89.5)
Diabetes, *n* (%)	60 (26.2)
AF, *n* (%)	36 (15.7)
PAD, *n* (%)	14 (6.1)
Previous PCI, *n* (%)	84 (63.3)
Previous CABG, *n* (%)	6 (2.6)
CKD (<30 ml/min), *n* (%)	48 (20.9)
Angina, *n* (%)	77 (68.1)
CCS, *n* (%)	114 (49.8)
ACS, *n* (%)	115 (50.2)
Three-vessel coronary artery disease, *n* (%)	68 (29.7)
Procedural variables	
Calcified stenosis, *n* (%)	145 (66.8)
Functional evaluation (FFR/RFR), *n* (%)	64 (27.9)
Intracoronary imaging (IVUS), *n* (%)	25 (10.9)
Medina (1,1,1), *n* (%) Provisional technique DK crush technique	66 (30.7) 45 21
Insertion of 1 DES, *n* (%)	110 (48)
Insertion of 2 DES, *n* (%)	64 (27.9)
Mechanical support, *n* (%) Intra-aortic balloon pump Impella device	20 (8.7) 18 2

ACS, acute coronary syndrome; AF, atrial fibrillation; CABG, coronary artery bypass grafting; CCS, chronic coronary syndrome; CKD, chronic kidney disease; DES, drug-eluting stent; FFR, fractional flow reserve; IVUS, intravascular ultrasound; LVEF, left ventricular ejection fraction; PAD, peripheral artery disease; PCI, percutaneous coronary intervention; RFR, resting flow reserve.

A significant proportion of patients had a history of prior PCI (63.3%), and 6.1% were diagnosed with PAD. The indication for the procedure was chronic coronary syndrome in 114 patients (49.8%), who were all evaluated by a dedicated Heart Team, which considered clinical, anatomical, and procedural factors before opting for PCI as the preferred strategy, and ACS in 115 patients (50.2%).

From an angiographic perspective, 66.8% of patients exhibited calcified stenosis of the LMCA at the time of the index procedure, while 29.7% had three-vessel coronary artery disease. According to the Medina classification of the LMCA stenosis, 66 (30.7%) patients were categorized as 1,1,1, indicating significant bifurcation involvement. Among these, 45 patients were treated using the provisional stenting technique, while 21 patients underwent DK crush stenting.

Procedurally, a single DES was used in 48% of cases, while 41.9% of patients received multiple DES, predominantly two stents (*n* = 64). Additionally, functional intracoronary assessment using FFR and resting full-cycle ratio was performed in 27.9% of cases, while intracoronary imaging via IVUS was utilized in 10.9% of patients.

Mechanical support during the procedure was required in 20 (8.7%) cases, with 18 patients managed with intra-aortic balloon pump support and two patients with the Impella device.

### In-stent restenosis

The primary endpoint of the study was the identification of ISR during the 8-month angiographic follow-up. Among the 229 patients who completed the angiographic follow-up, 24 (10.5%) developed ISR. Seventeen patients undergoing angiographic follow-up were completely asymptomatic, while the remaining seven presented with angina symptoms.

Of the symptomatic patients, four presented with CCS grade 1 angina, three with CCS grade 2, and none with CCS grades 3 or 4. The diagnosis of restenosis was established using various methods: in 16 (66.7%) cases, it was confirmed via FFR; in four cases (16.7%), through intravascular ultrasound (IVUS); and in the remaining four cases (16.7%), based on angiographic criteria.

All 24 patients with ISR underwent further treatment: 15 patients (62.5%) were treated with a drug-eluting balloon, one patient (4.2%) received a DES, and eight patients (33.3%) underwent surgical revascularization via CABG (Table 2).

**Table 2:** Diagnostic tools and treatment of patients with in-stent restenosis

ID	Angina (1,0)^a^	CCS scale (0,1,2,3,4)	Diagnosis ISR (FFR, IVUS, angiographic)	TLR
BS-001	0		FFR	DEB
BS-002	1	1	FFR	DEB
BS-003	0		FFR	DEB
BS-004	1	2	Angiographic	DEB
BS-005	0		FFR	CABG
BS-006	0		FFR	CABG
BS-007	0		FFR	DEB
BS-008	1	2	FFR	DEB
BS-009	1	2	FFR	DEB
BS-010	0		IVUS	DEB
BS-011	0		FFR	DEB
BS-012	0		Angiographic	CABG
BS-013	0		IVUS	DEB
BS-014	1	1	Angiographic	DES
BS-015	0		FFR	DEB
BS-016	0		FFR	CABG
BS-017	0		FFR	CABG
BS-018	0		IVUS	DEB
BS-019	0		FFR	CABG
BS-020	1	1	Angiographic	DEB
BS-021	0		FFR	CABG
BS-022	0		Angiographic	DEB
BS-023	1	1	FFR	DEB
BS-024	0		FFR	CABG

CABG, coronary artery bypass grafting; CCS, chronic coronary syndrome; DEB, drug-eluting balloon; FFR, fractional flow reserve; ISR, in-stent restenosis; IVUS, intravascular ultrasound; TLR, target lesion revascularization.

a Angina assessment at angiographic follow-up.

### Predictors of in-stent restenosis: results of univariate and multivariate analysis

The univariate analysis identified several significant predictors of ISR (Table 3). Specifically, CKD emerged as a strong risk factor, with an odds ratio of 3.84 (*P* = 0.003), indicating that patients with CKD are nearly four times more likely to develop restenosis. Similarly, diabetes was associated with an odds ratio of 3.18 (*P* = 0.008), suggesting that diabetic patients have more than three times the likelihood of restenosis compared with nondiabetic patients.

**Table 3:** Univariate and multivariate analysis identifying clinical and procedural predictors of in-stent restenosis

Variables	Odds ratio	*P* value	Confidence interval
Univariate analysis			
Age	0.98	0.344	0.95–1.02
Sex (male)	0.42	0.055	0.17–1.02
CKD (<30 ml/min)	3.84	**0.003**	1.59–9.25
BMI	0.99	0.767	0.94–1.05
AF	1.08	0.893	0.35–3.37
Diabetes	3.18	**0.008**	1.34–7.53
PAD	1.34	0.710	0.28–6.34
LVEF	0.99	0.546	0.95–1.02
ACS	0.38	0.038	0.15–0.95
Calcified stenosis	0.99	0.986	0.40–2.44
Medina 1,1,1	1.90	0.147	0.79–4.53
2 DES	0.60	0.181	0.75–4.44
Angina^a^	2.49	0.063	0.95–6.55
Multivariate analysis			
CKD (<30 ml/min)	2.71	**0.037**	1.06–6.94
Diabetes	2.54	**0.047**	1.01–6.41
ACS	0.40	0.062	0.15–1.04

Bold values indicate the risk factors that were statistically significant predictors of in-stent restenosis in both univariate and multivariate analyses.

ACS, acute coronary syndrome; AF, atrial fibrillation; CKD, chronic kidney disease; DES, drug-eluting stent; LVEF, left ventricular ejection fraction; PAD, peripheral artery disease.

a Angina refers to symptoms reported at the time of angiographic follow-up.

Conversely, presentation with ACS was found to be a protective factor, with an odds ratio of 0.38 (*P* = 0.038), indicating a reduced likelihood of restenosis in these patients. Multivariate analysis confirmed CKD and diabetes as independent predictors of ISR.

### Survival analysis of the study population

**Fig. 2:**
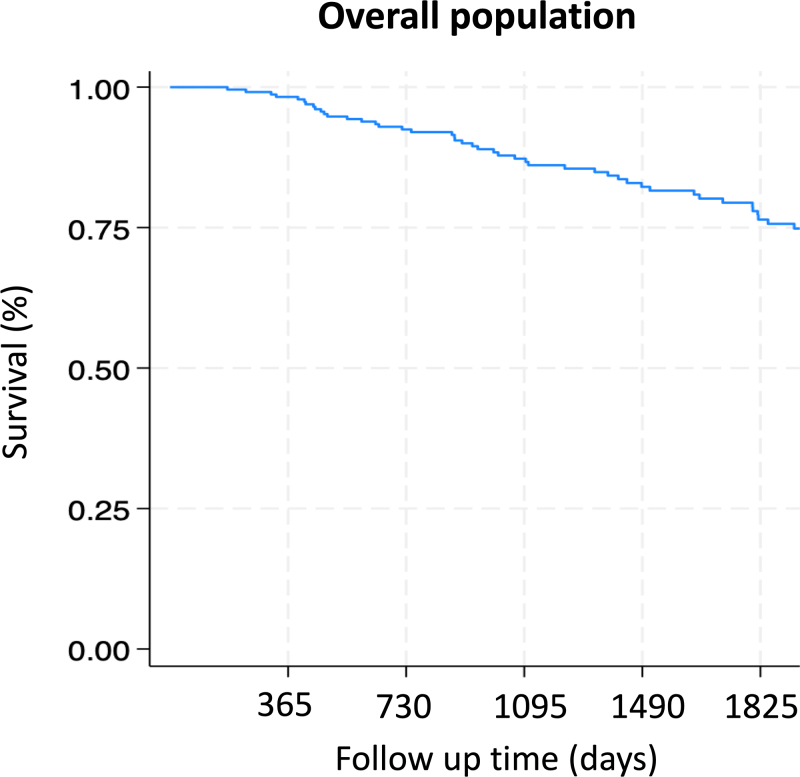
Kaplan–Meier survival curve depicting overall survival of the study population.

The findings of our study indicate that coronary angiography is a safe procedure. Among the 229 patients who underwent angiographic follow-up 8 months after stent implantation in the LMCA, no major complications were reported, including death, clinically significant stroke, iatrogenic coronary dissection, major periprocedural bleeding (Bleeding Academic Research Consortium 2 to 5), or periprocedural myocardial infarction. Additionally, no patients required continuous venovenous hemofiltration due to acute kidney injury, following angiography. Regarding overall survival in the study population, the follow-up revealed a median duration of 4.5 years (interquartile range: 2.5–6.8 years). Survival rates were as follows: 12 months after angioplasty, survival was notably high at 97.7% (95% CI: 95.8–99.7%). At 2 years, the survival rate was 92.2% (95% CI: 88.7–95.8%), and at 4 years, it was 81.5% (95% CI: 76.0–87.3%) (Fig. 2).

### Subanalysis: patients with diabetes and chronic kidney disease

**Fig. 3:**
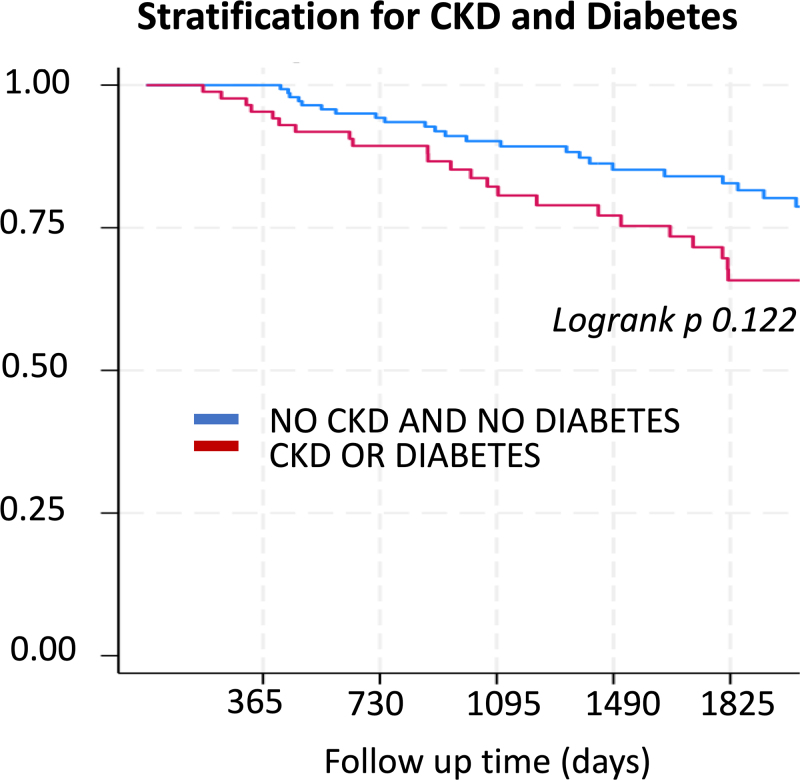
Kaplan–Meier survival curve comparing patients with at least one cardiovascular risk factor (diabetes or advanced chronic kidney disease, defined as GFR < 30 ml/min) vs. patients without these risk factors. GFR, glomerular filtration rate.

A subanalysis was performed, dividing the population into two groups: patients with at least one of the following cardiovascular risk factors – diabetes or advanced CKD [glomerular filtration rate (GFR) < 30 ml/min) (*n* = 86) – and patients without these risk factors (*n* = 143). As depicted in the Kaplan–Meier survival curve (Fig. 3), although the mortality difference between the two groups did not reach statistical significance (LogRank *P* = 0.122), the survival curves suggest a trend toward higher mortality in patients with diabetes or CKD. While not statistically significant, this result highlights a potential association between these risk factors and increased mortality in our cohort of patients with LMCA stenosis.

### Subanalysis: patients with acute and chronic coronary syndromes

A further subanalysis was conducted, dividing the population into two groups based on the reason for admission: ACS and CCS. In total, 115 patients were classified under the ACS group, while 114 were categorized in the CCS group. Analysis of these subpopulations revealed several significant differences. Notably, the median left ventricular ejection fraction was higher in patients with CCS compared with those with ACS (58% vs. 45%, *P* < 0.001). Furthermore, dyslipidemia was more prevalent in the CCS group (93.9% vs. 85.2%, *P* = 0.033). Conversely, patients with CCS exhibited a lower incidence of CKD with a GFR <30 ml/min (16.7% vs. 25.2%), and the need for mechanical support was significantly less frequent in the CCS group (0.8% vs. 16.5%). For CCS patients, angina severity was assessed using the CCS classification. Among the 114 CCS patients, 1.8% had CCS grade 1, 28.9% CCS grade 2, 18.2% CCS grade 3, and 17.5% CCS grade 4.

**Fig. 4:**
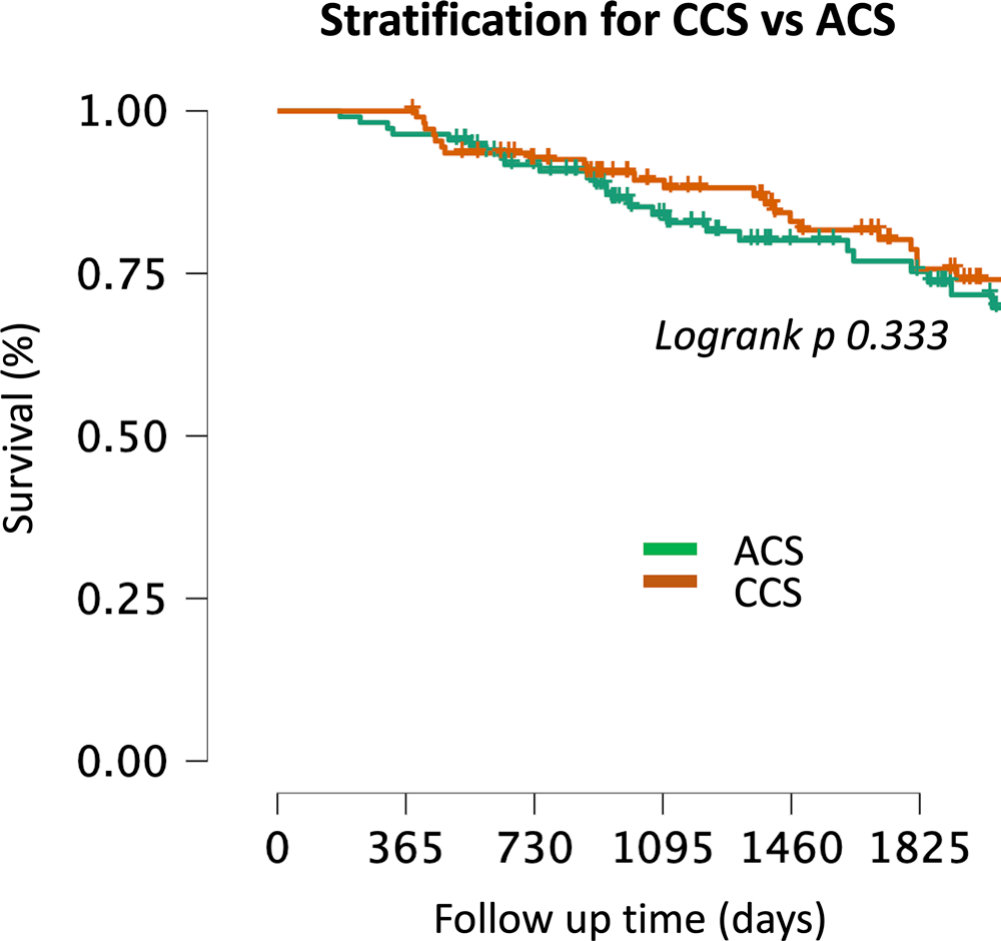
Kaplan–Meier survival curve stratified by the primary reason for admission: chronic coronary syndrome (CCS) vs. acute coronary syndrome (ACS).

Finally, survival curves stratified by admission modality (ACS vs. CCS) showed no substantial differences between the two groups. As illustrated in Fig. 4, there were no significant variations in survival between ACS and CCS patients (LogRank *P* = 0.333).

## Discussion

### In-stent restenosis rate

In this study, we observed an ISR rate of 10.5% among patients undergoing LMCA PCI with stenting, based on angiographic follow-up at 8 months. This confirms that ISR remains a significant complication in unprotected LMCA interventions. Considering the critical role of the LMCA in myocardial perfusion, undiagnosed or untreated ISR can have catastrophic outcomes. Data from observational registries highlight the value of intravascular imaging (IVUS or optical coherence tomography) during LMCA revascularization. For instance, the British Cardiovascular Intervention Society registry demonstrated significantly reduced mortality at 30 days (2.9% vs. 6.6%) and 12 months (9.0% vs. 15.5%) when IVUS was employed compared with angiography alone [13]. Similarly, the Swedish Coronary Angiography and Angioplasty Registry showed lower ISR rates (5.3% vs. 2.4%) and reduced stent thrombosis and mortality over 10 years in IVUS-guided procedures [14]. The limited use of intravascular imaging (10.9%) in our cohort may partly explain the relatively high ISR rate observed. Nevertheless, our results align with prior studies, such as a retrospective analysis reporting an ISR rate of 9.7% in LMCA PCI [15]. These findings underscore that ISR remains a clinical challenge, even with advancements in stent technology and imaging. Systematic angiographic surveillance in the medium term remains a reliable and effective strategy for early ISR detection and management.

### Predictors of in-stent restenosis

Our results confirm that CKD and diabetes are independent predictors of ISR, consistent with previous studies [16]. Patients with CKD showed a nearly fourfold increased risk of ISR, while patients with diabetes had a threefold increased risk. Despite the exclusive use of second-generation DES in this study, ISR remains a concern for patients with these comorbidities. Neoatherosclerosis, a phenomenon associated with earlier onset in DES, likely contributes to ISR, particularly in patients with CKD or smoking history [17]. Aggressive follow-up strategies and optimized medical therapy are essential to mitigate this risk in high-risk populations.

### Asymptomatic in-stent restenosis

A notable finding was that only 29.2% of patients with ISR were symptomatic at angiographic follow-up. Despite reporting symptoms, none of the seven symptomatic patients sought earlier evaluation, likely due to mild or underreported symptoms. This underscores the limitations of relying on symptoms, such as angina, as reliable indicators of ISR in LMCA PCI patients. Silent restenosis can lead to severe complications if undetected [9,18]. Noninvasive testing, including myocardial scintigraphy, may yield false negatives in LMCA PCI patients due to uniform signal attenuation caused by the involvement of a large myocardial territory. Thus, angiographic follow-up might play a valuable role in identifying asymptomatic ISR and mitigating the risk of adverse outcomes.

### Limitations

This study is limited by its retrospective, single-center design, which may restrict the generalizability of the findings. Variations in patient demographics, operator experience, and procedural protocols across institutions and healthcare systems could influence outcomes. Nevertheless, as all included patients underwent angiographic follow-up as part of an internal protocol, procedural decision-making and the intended initial strategy were similar across the cohort. These insights contribute to the growing body of evidence on optimizing outcomes in LMCA PCI patients. Our results should be interpreted within the context of broader clinical practice, reinforcing the need for multicenter validation in diverse real-world populations.

Additionally, the limited use of intravascular imaging, with only 10.9% of patients undergoing IVUS evaluation, may have influenced our findings and potentially overestimated the incidence of ISR. Given the retrospective nature of the study, with procedures performed since 2013, the use of IVUS has, however, progressively increased and is now more widely adopted in contemporary practice, as recommended by current guidelines.

Despite these limitations, our findings reinforce the importance of systematic angiographic follow-up in detecting ISR, particularly given that a significant proportion of patients were asymptomatic at the time of diagnosis. The absence of a control group without systematic angiographic follow-up limits our ability to assess whether this approach improves long-term outcomes. A comparative analysis could have provided more definitive conclusions on the efficacy of angiographic surveillance in reducing adverse events.

### Future directions

Future research should aim to clarify the most effective follow-up strategy after PCI for unprotected LMCA disease, particularly in high-risk patients with CKD or diabetes. Randomized controlled trials comparing systematic angiographic follow-up (with physiology-guided assessment for intermediate lesions) vs. standard clinical follow-up could determine whether proactive imaging surveillance reduces adverse events in this vulnerable population. Such studies would help define whether routine anatomical reassessment confers tangible clinical benefit beyond symptom-driven care and may guide a more personalized approach to post-PCI management.

## Conclusions

In this study, we observed a 10.5% incidence of ISR following LMCA PCI, with CKD and diabetes identified as independent predictors. Importantly, the majority of ISR cases were asymptomatic, highlighting the limitations of symptom-based detection and the potential unreliability of noninvasive imaging in this population. Systematic angiographic follow-up proved to be a critical tool for early detection and management of ISR, especially in high-risk patients, reducing the likelihood of severe complications. Despite advances in stent technology and imaging, ISR remains a significant challenge, underscoring the need for optimized follow-up strategies and tailored therapy in LMCA PCI patients.

## Acknowledgements

### Conflicts of interest

M.A. has received speaker fees from Abbott Vascular and Edwards Lifesciences. M. Metra has received honoraria for participation in steering committees or advisory boards or for speeches from Abbott Vascular, Amgen, AstraZeneca, Bayer, Edwards, Fresenius, Novartis, and Servier, outside of the submitted work. For the remaining author, there are no conflicts of interest.

## Supplementary Material

## References

[1] Lee PH Ahn JM Chang M Baek S Yoon S-H Kang S-J. Left main coronary artery disease: secular trends in patient characteristics, treatments, and outcomes. J Am Coll Cardiol 2016; 68:1233–1246. 27609687 10.1016/j.jacc.2016.05.089

[2] Vrints C Andreotti F Koskinas KC Rossello X Adamo M Ainslie J; ESC Scientific Document Group. 2024 ESC Guidelines for the management of chronic coronary syndromes. Eur Heart J 2024; 45:3415–3537. 39210710 10.1093/eurheartj/ehae177

[3] Stone GW Sabik JF Serruys PW Simonton CA Généreux P Puskas J; EXCEL Trial Investigators. Everolimus-eluting stents or bypass surgery for left main coronary artery disease. N Engl J Med 2016; 375:2223–2235. 27797291 10.1056/NEJMoa1610227

[4] Chieffo A Meliga E Latib A Park S-J Onuma Y Capranzano P. Drug-eluting stent for left main coronary artery disease. The DELTA registry: a multicenter registry evaluating percutaneous coronary intervention versus coronary artery bypass grafting for left main treatment. JACC Cardiovasc Interv 2012; 5:718–727. 22814776 10.1016/j.jcin.2012.03.022

[5] Chieffo A Tanaka A Giustino G Briede I Sawaya FJ Daemen J; DELTA 2 Investigators. The DELTA 2 registry: a multicenter registry evaluating percutaneous coronary intervention with new-generation drug-eluting stents in patients with obstructive left main coronary artery disease. JACC Cardiovasc Interv 2017; 10:2401–2410. 29217002 10.1016/j.jcin.2017.08.050

[6] Tarantini G Fovino LN Varbella F Trabattoni D Caramanno G Trani C. A large, prospective, multicentre study of left main PCI using a latest-generation zotarolimus-eluting stent: the ROLEX study. EuroIntervention 2023; 18:e1108–e1119. 36043326 10.4244/EIJ-D-22-00454 PMC9909455

[7] Kirschbaum SW de Feyter PJ van Geuns RJ. Cardiac magnetic resonance imaging in stable ischaemic heart disease. Neth Heart J 2011; 19:229–235. 21487751 10.1007/s12471-011-0106-4 PMC3087021

[8] Sheiban I Moretti C D’Ascenzo F Chieffo A Taha S Connor SO. Long-term (≥10 years) safety of percutaneous treatment of unprotected left main stenosis with drug-eluting stents. Am J Cardiol 2016; 118:32–39. 27209125 10.1016/j.amjcard.2016.04.007

[9] Cassese S Byrne RA Schulz S Hoppman P Kreutzer J Feuchtenberger A. Prognostic role of restenosis in 10 004 patients undergoing routine control angiography after coronary stenting. Eur Heart J 2015; 36:94–99. 25298237 10.1093/eurheartj/ehu383

[10] Kuntz RE Baim DS. Defining coronary restenosis. Newer clinical and angiographic paradigms. Circulation 1993; 88:1310–1323. 8353892 10.1161/01.cir.88.3.1310

[11] Tonino PA De Bruyne B Pijls NH Siebert U Ikeno F van' t Veer M; FAME Study Investigators. Fractional flow reserve versus angiography for guiding percutaneous coronary intervention. N Engl J Med 2009; 360:213–224. 19144937 10.1056/NEJMoa0807611

[12] Kang SJ Ahn JM Song H Kim W-J Lee J-Y Park D-W. Comprehensive intravascular ultrasound assessment of stent area and its impact on restenosis and adverse cardiac events in 403 patients with unprotected left main disease. Circ Cardiovasc Interv 2011; 4:562–569. 22045969 10.1161/CIRCINTERVENTIONS.111.964643

[13] Kinnaird T Johnson T Anderson R Gallagher S Sirker A Ludman P. Intravascular imaging and 12-month mortality after unprotected left main stem PCI: an analysis from the British Cardiovascular Intervention Society database. JACC Cardiovasc Interv 2020; 13:346–357. 32029252 10.1016/j.jcin.2019.10.007

[14] Andell P Karlsson S Mohammad MA Götberg M James S Jensen J. Intravascular ultrasound guidance is associated with better outcome in patients undergoing unprotected left main coronary artery stenting compared with angiography guidance alone. Circ Cardiovasc Interv 2017; 10:e004813. 28487356 10.1161/CIRCINTERVENTIONS.116.004813

[15] Sheiban I Sillano D Biondi-Zoccai G Chieffo A Colombo A Vecchio S. Incidence and management of restenosis after treatment of unprotected left main disease with drug-eluting stents: 70 restenotic cases from a cohort of 718 patients: FAILS (Failure in Left Main Study). J Am Coll Cardiol 2009; 54:1131–1136. 19761932 10.1016/j.jacc.2009.06.018

[16] Iqbal J Serruys PW Silber S Kelbaek H Richardt G Morel M-A. Comparison of zotarolimus- and everolimus-eluting coronary stents: final 5-year report of the RESOLUTE all-comers trial. Circ Cardiovasc Interv 2015; 8:e002230. 26047993 10.1161/CIRCINTERVENTIONS.114.002230 PMC4495878

[17] Otsuka F Byrne RA Yahagi K Mori H Ladich E Fowler DR. Neoatherosclerosis: overview of histopathologic findings and implications for intravascular imaging assessment. Eur Heart J 2015; 36:2147–2159. 25994755 10.1093/eurheartj/ehv205

[18] Jakobsen L Christiansen EH Freeman P Kahlert J Veien K Maeng M. Comparison of outcome after percutaneous coronary intervention for de novo and in-stent restenosis indications. Am J Cardiol 2025; 235:1–8. 39461401 10.1016/j.amjcard.2024.10.019

