# Supplementary material for: Eight-month angiographic outcomes and in-stent restenosis in patients undergoing percutaneous coronary intervention on unprotected left main coronary artery
Source: Coron Artery Dis. 2025 Oct 24;37(1):56–62. doi: 10.1097/MCA.0000000000001557 (PMC12673903; doi:10.1097/MCA.0000000000001557)
Supplement: Supplementary file 1 [file cad-37-56-s001.docx]

**Supplementary Table 1.** Baseline characteristics and the reasons for exclusion from angiographic follow up of 68 patients

| ID | Sex (M; F) | Age | Admission  (ACS=1; CCS=2) | Reason not perfomed angiographic follow up |
| --- | --- | --- | --- | --- |
| 1 | M | 62 | 1 | Refused angiographic follow-up |
| 2 | M | 82 | 1 | Refused angiographic follow-up |
| 3 | F | 85 | 2 | Frailty, follow-up on a clinical basis |
| 4 | F | 81 | 1 | Death during the index hospitalization (cardiogenic shock) |
| 5 | F | 83 | 2 | Refused angiographic follow-up |
| 6 | M | 68 | 1 | Refused angiographic follow-up |
| 7 | F | 89 | 1 | Frailty, follow-up on a clinical basis |
| 8 | M | 84 | 1 | Death during the index hospitalization (infection) |
| 9 | M | 82 | 2 | Refused angiographic follow-up |
| 10 | F | 68 | 1 | Refused angiographic follow-up |
| 11 | M | 85 | 2 | Frailty, follow-up on a clinical basis |
| 12 | M | 68 | 2 | Refused angiographic follow-up |
| 13 | M | 60 | 1 | Refused angiographic follow-up |
| 14 | F | 85 | 1 | Frailty, follow-up on a clinical basis |
| 15 | M | 81 | 2 | Refused angiographic follow-up |
| 16 | M | 83 | 2 | Refused angiographic follow-up |
| 17 | M | 88 | 2 | Frailty, follow-up on a clinical basis |
| 18 | M | 57 | 1 | Refused angiographic follow-up |
| 19 | M | 85 | 2 | Frailty, follow-up on a clinical basis |
| 20 | F | 81 | 2 | Refused angiographic follow-up |
| 21 | F | 60 | 1 | Refused angiographic follow-up |
| 22 | M | 88 | 1 | Frailty, follow-up on a clinical basis |
| 23 | F | 83 | 1 | Death during the index hospitalization (cardiogenic shock) |
| 24 | M | 91 | 1 | Frailty, follow-up on a clinical basis |
| 25 | F | 85 | 1 | Frailty, follow-up on a clinical basis |
| 26 | M | 92 | 1 | Frailty, follow-up on a clinical basis |
| 27 | M | 62 | 2 | Refused angiographic follow-up |
| 28 | M | 88 | 1 | Frailty, follow-up on a clinical basis |
| 29 | F | 81 | 2 | Refused angiographic follow-up |
| 30 | F | 94 | 2 | Frailty, follow-up on a clinical basis |
| 31 | M | 57 | 1 | Refused angiographic follow-up |
| 32 | M | 66 | 2 | Refused angiographic follow-up |
| 33 | F | 62 | 1 | Refused angiographic follow-up |
| 34 | M | 73 | 1 | Death during the index hospitalization (cardiogenic shock) |
| 35 | M | 90 | 1 | Frailty, follow-up on a clinical basis |
| 36 | M | 84 | 2 | Refused angiographic follow-up |
| 37 | M | 73 | 1 | Refused angiographic follow-up |
| 38 | M | 85 | 2 | Frailty, follow-up on a clinical basis |
| 39 | F | 81 | 1 | Refused angiographic follow-up |
| 40 | M | 81 | 1 | Refused angiographic follow-up |
| 41 | M | 85 | 2 | Frailty, follow-up on a clinical basis |
| 42 | M | 84 | 2 | Refused angiographic follow-up |
| 43 | M | 55 | 1 | Refused angiographic follow-up |
| 44 | M | 83 | 1 | Death during the index hospitalization (infection) |
| 45 | M | 58 | 2 | Refused angiographic follow-up |
| 46 | M | 74 | 2 | Refused angiographic follow-up |
| 47 | F | 86 | 2 | Frailty, follow-up on a clinical basis |
| 48 | M | 82 | 1 | Death during the index hospitalization (infection) |
| 49 | M | 86 | 2 | Frailty, follow-up on a clinical basis |
| 50 | M | 83 | 1 | Death during the index hospitalization (infection) |
| 51 | M | 92 | 2 | Frailty, follow-up on a clinical basis |
| 52 | F | 84 | 1 | Refused angiographic follow-up |
| 53 | M | 82 | 1 | Refused angiographic follow-up |
| 54 | F | 84 | 2 | Refused angiographic follow-up |
| 55 | M | 82 | 1 | Refused angiographic follow-up |
| 63 | M | 72 | 1 | Death during the index hospitalization (cardiogenic shock) |
| 57 | M | 70 | 1 | Refused angiographic follow-up |
| 58 | M | 57 | 1 | Death during the index hospitalization (infection) |
| 59 | F | 91 | 1 | Frailty, follow-up on a clinical basis |
| 60 | F | 92 | 1 | Frailty, follow-up on a clinical basis |
| 61 | M | 60 | 2 | Refused angiographic follow-up |
| 62 | M | 81 | 1 | Refused angiographic follow-up |
| 63 | M | 72 | 1 | Death during the index hospitalization (cardiogenic shock) |
| 64 | M | 73 | 2 | Refused angiographic follow-up |
| 65 | M | 84 | 2 | Refused angiographic follow-up |
| 66 | F | 85 | 1 | Frailty, follow-up on a clinical basis |
| 67 | M | 84 | 1 | Refused angiographic follow-up |
| 68 | F | 86 | 1 | Refused angiographic follow-up |
